# Drug Utilisation Review among Geriatric Patients with Noncommunicable Diseases in a Primary Care Setting in Malaysia

**DOI:** 10.3390/healthcare11121665

**Published:** 2023-06-06

**Authors:** Priya Manirajan, Palanisamy Sivanandy

**Affiliations:** Department of Pharmacy Practice, School of Pharmacy, International Medical University, Kuala Lumpur 57000, Malaysia; priyamanirajan@imu.edu.my

**Keywords:** elderly, prescription, multimorbidity, disease, age, aging, medications, polypharmacy

## Abstract

A prospective cross-sectional study was conducted to analyse the drugs prescribed to the elderly population with noncommunicable diseases and to determine the polypharmacy at a primary care clinic in Negeri Sembilan, Malaysia. The study was conducted for 6 months at the primary care clinic of Gemas. Geriatric patients above the age of 65 and diagnosed with noncommunicable diseases were included upon providing written informed consent. The majority of the geriatric patients were between 65 and 69 years (mean: 69.72 ± 2.85) and prescribed 4 or more medications (mean: 5.18 ± 0.64, *p* = 0.007). More than 95% (*n* = 295) of the geriatrics were found to have multimorbidity, in which around 45% (*n* = 139) had type-2 diabetes together with hypertension and dyslipidaemia. Combination therapy was prescribed to more than 97% (*n* = 302) of the elderly, whereas cardiovascular and endocrine medications were the most commonly prescribed. Ten prescriptions were found to have drug-related problems, prescribing cascade (80%), lack of medicine optimisation (10%), and inappropriate prescription (10%). In this study, the majority of the elderly had multimorbidity; polypharmacy was commonly seen among geriatric patients. Polypharmacy is the biggest threat to the elderly population, as it increases the chances of falls and fall-related injuries. Medicine optimisation and deprescribing will reduce the chances of drug-related problems and morbidity and mortality associated with polypharmacy and over-consumption of medications. Hence, the study recommends the health fraternity look for medication optimisation and deprescribing to reduce the future complications associated with polypharmacy.

## 1. Introduction

As a person’s age increases, a decline in physiological function is observed, which is one of the contributing factors to the development of noncommunicable diseases. The presence of a disease interrupts metabolic homeostasis and inflammatory status leading to the cascade development of multimorbidities [1,2,3]. This is true in the case of elderly patients identified with metabolic syndrome whereby unhealthy lifestyle, dyslipidaemia, glucose intolerance, hypertension, proinflammatory state, and prothrombic state increase the risk of developing stroke [4]. According to the world disease burden data, more than 60% of the burden of disease results from noncommunicable diseases (NCDs). Nevertheless, the rates of disease burden and disability-adjusted life year (DALYs) rates in people over 70 years old remain significantly high [5,6]. Elderlies living with multimorbidities are subjected to multiple prescription medications due to treatment complexity.

With widely accepted, polypharmacy is defined as consuming five or more different medications a patient at a given time, whereas ten or more medication intake is defined as excessive polypharmacy [7,8,9,10,11]. Polypharmacy among the elderly population is of concern as it adversely affects their health outcomes. Multiple medication intake coupled with increased drug frequencies and special instructions burdens elderly patients as it affects their adherence to pharmacotherapy [12,13]. In addition, polypharmacy has been associated with adverse drug reactions in the elderly population due to plausible drug–drug interactions, drug-disease interactions, decreased rate of drug clearance which prolongs the duration of drug retention in the body, and all leading hospitalisations [14,15]. Polypharmacy, in most cases, adversely affects geriatric patients’ quality of life; however, in certain cases, it is needed to prolong one’s life as well.

The prevalence of polypharmacy is expected to rise with the increasing global elderly population, including in Malaysia, whereby the population is expected to rise from 7.2% to 14.5% in 2040 [16]. This leads to increased drug-related problems among the elderly population in the country. Hence, a drug utilisation review can be conducted to determine sensible drug use in this population. Drug utilisation review (DUR) or drug utilisation evaluation is defined as the marketing, distribution, prescription, and use of drugs in a society, with special emphasis on the resulting medical, social, and economic consequences. This definition was created by the WHO back in 1977 [17]. Since then, there have been multiple definitions for DUR that were observed. Thadanki et al. defined DUR as an authorised, structured, ongoing review of prescribing, dispensing, and use of medications [18]. A DUR study conducted in Malaysia provided a similar definition stating that an authorised, structured, and ongoing review of healthcare providers’ prescribing patterns, pharmacists’ dispensing patterns, and patients’ use of medication [19]. Most of the studies investigating DUR are typically conducted in hospital settings [20,21,22,23], but not many focusing the primary care setting, even in other Asian countries. By employing the DUR, this study aims to determine the prevalence of polypharmacy among the elderly population in a primary care setting, drug prescribing patterns for NCDs, and to determine the possible drug-related problems in this population.

## 2. Materials and Methods

### 2.1. Study Design, Setting, and Population

This is a cross-sectional study that uses descriptive DUR to address the objectives of this study. The study was carried out for six months in a primary care setting situated in the rural region of Negeri Sembilan, which is Gemas. The calculated sample size for this study was 310. The study comprised participants aged 65 and above who were seeking medical treatment at this clinic. The participants who were diagnosed with chronic diseases with long-term medications were included in the study. Patients with visual or hearing impairments were not included in the study.

### 2.2. Study Instrument and Data Collection

A data collection sheet was used to collect information regarding sociodemographic details as well as the participant’s diagnosis and medication history. The respondent’s sociodemographic information, such as age, gender, ethnicity, marital status, and education level, were gathered. Patients’ medical reports were used to collect comorbidities and long-term medication data. When medical records were unavailable, diagnosis cards, doctors’ notes, clinic records, and participant self-explanations were utilised. Only the diagnosis was recorded, and for medication, the type and number of medications consumed were recorded. Respondents who met the inclusion criteria and were willing to participate in the study were recruited. Informed written consent was obtained from each participant prior to enrolling in the study. Each participant’s medical records were reviewed prospectively.

### 2.3. Statistical Analysis

The collected data was analysed descriptively and reported as mean, standard deviation (SD), or frequency (%). The collected data were tabulated and analysed in terms of the study’s objectives using inferential and descriptive statistics using Statistical Package for the Social Sciences (SPSS).

### 2.4. Ethical Considerations

The International Medical University’s research and ethics committee (approval number: MPP1-2021(09)) and the head of the study facility in Malaysia provided ethical permission for this study. Throughout the study, the confidentiality of all information acquired during the data collection process was maintained.

## 3. Results

### 3.1. Respondents’ Demographic Characteristics

During the study period, 329 elderly patients visited the primary clinic, whereas 310 participants agreed to participate in this study by providing written informed consent. Out of 310 participants, 55% of them were females, slightly higher in terms of gender. The majority of the respondents fall between the age category of 65 years and 69 years. This was followed by 104 respondents within the age category of 70 to 74 years. In terms of ethnicity, 308 participants were Malay, while two participants were of Indian ethnicity. Among them, 74% obtained only primary-level education. The demographic characteristics of the respondents are presented in Table 1.

### 3.2. Respondents’ Comorbidity Status

Among the respondents, 44.84% of them had three types of NCDs: type 2 diabetes mellitus (T2DM), hypertension, and dyslipidaemia. Around 87 respondents were diagnosed with hypertension and dyslipidaemia. The majority of the respondents have been diagnosed with at least one comorbidity. Only 15 respondents (4.84%) were diagnosed with a single chronic disease which are hypertension, dyslipidaemia, chronic obstructive pulmonary disease (COPD), and myocardial infarction (MI), while the remaining patients (*n* = 295; 95.16%) have multimorbidity. Twenty-one respondents (6.77%) have been identified to be diagnosed with four types of NCDs. Out of 310 respondents, 284 of them (91.61%) were diagnosed with hypertension, 282 (90.96%) were diagnosed with dyslipidaemia, and 186 (60%) were diagnosed with T2DM. The details are presented in Table 2.

### 3.3. Medication Prescribed to the Respondents

In this study, the details of medications prescribed to elderly patients were collected from their medication records, prescriptions, and case notes. The medications were then categorised into monotherapy and combination therapy, class of drugs, and later the number of medications received by the patients was arranged chronologically to find out how many medications the patients were currently receiving. The majority of the respondents (*n* = 302; 97.42%) were prescribed combination pharmacotherapy, while eight respondents (2.58%) were prescribed a single medication. Overall, a total of 245 drug combinations have been identified in this study. The details of the prescribed medications are presented in Table 3. Among the combinations prescribed, 1 respondent (0.33%) was prescribed the highest number of medications, which was 10, followed by 6 respondents (1.99%) with 9 medications, 13 respondents (4.30%) with 8 medications, and 23 respondents (7.62%) with 7 medications. Most of the respondents (*n* = 69; 22.85%) were prescribed four types of medications. Around 230 (74.19%) respondents’ prescriptions were detected with polypharmacy. The mean number of drugs prescribed to respondents was 5.18 ± 0.64. There is a significant difference in the number of medications prescribed to the respondents (*p* = 0.007). The prevalence of polypharmacy in this group was 51%, which means half of the study population is consuming five or more long-term medications. Polypharmacy is majorly detected with antihypertensive medications. The details of the number of prescribed medications are presented in Table 4. The frequency of individual medication prescribed to the respondents is presented in Table 5.

### 3.4. Drug-Related Problems

The drug-related problems were screened using the prescription given to the study respondents. There were a few drug-related problems observed, which are preventable in nature. Antihypertensives and diuretics, either alone or in combination, were commonly prescribed among the geriatric patients in the present study, followed by antidiabetic and other medications. Antihypertensives, diuretics, and antidiabetics have the potential to cause low blood pressure, impairment of balance or gait performance, and low blood sugar in geriatric patients, which tend to cause falls and fall-related injuries. The details of drug-related problems are presented in Table 6.

## 4. Discussion

The present study aimed to assess the pattern of drugs prescribed to the elderly population in a primary care clinic in the rural area of Gemas, a backward area of the state of Negeri Sembilan. The majority of the population in this area has an average to moderate level of living style and typical domestic culture of heritage and food culture. Females were more prevalent than males, and the majority were between 65 and 69 years old. Populations in Asia, especially in developed economies, are rapidly ageing, which increases the prevalence of frailty and dependency, leading to an increase in complex health and social needs because an extended lifespan is not always accompanied by a prolongation of health span [24]. The ALLHAT trial conducted among 23,964 participants revealed that the mean age of the participants was 69.8 ± 6.8 years, and the study identified 267 falls, 249 orthostatic hypotension, and 755 syncopes [25].

Multimorbidity among the elderly population is a global concern. In the present study, almost 95% of the elderly patients had multiple disease conditions for a long period of time and are currently on multiple medications. Similarly, a recent study in China revealed that the prevalence of multimorbidity among old patients with chronic disease was 34.70%, and around 60.59% of the elderly had two coexisting chronic diseases [26]. A study conducted in the rural area, Tigiria block of Odisha, India, found that the rural population had a 48.8% overall prevalence of multimorbidity, and the multimorbidity was more common among females (50.4%) than males (47.4%) [27].

An 18-year population-based follow-up study conducted among the Finnish community-dwelling older people revealed that older adults aged 64.0 to 97.0 years (*n*  =  820; mean age 74.7 years) had frequent institutionalisation due to multimorbidity, the majority had five or more chronic conditions and mostly it is cerebrovascular oriented [28].

The 15 years follow-up SHARE (Survey of Health, Ageing, and Retirement in Europe) study concluded that the prevalence of multimorbidity among the elderly (mean age: 64.3 years) at baseline was 36.1%. At baseline, the presence of multimorbidity has significantly increased the risk of dementia among the overall study population (HR  =  1.14) and in individuals below 55 years (HR  =  2.06) [29].

### 4.1. Prevalence of Polypharmacy

Polypharmacy is a global health issue as the number of disease conditions among geriatrics increases the number of medications required for the management of illness. The prevalence of polypharmacy among older adults in the United States reported by physician offices from 2009 to 2016 was 36.8% [30]. The study also pointed out that the severity of polypharmacy was directly proportional to the number of chronic conditions a patient has. It has also been highlighted that 70.3% of patients with polypharmacy have been prescribed high-risk medications. The prevalence of polypharmacy across 17 European countries ranged from 26.3 to 39.9%, where Portugal, Israel, and the Czech Republic reported the highest prevalence [31]. The study reported that with increasing age, the prevalence of polypharmacy increases. In addition to this, lower quality of life, depression, and low income are associated with polypharmacy. A nationwide prevalence study in Spain from 2006 to 2014 reported that 21.9% of people receive polypharmacy [32]. Similarly, the prevalence increases with age. The prevalence of polypharmacy in Sweden increased from 16.9% in 2006 to 19.0% in 2014 [33]. This is probably due to the development of new guidelines, improvement in healthcare provided, and improvement in health literacy. The Malaysian Elders Longitudinal Research (MELoR) cohort study reported that the prevalence of polypharmacy among the elderly is 45.9% [34]. The study added 499 (86.6%) elderly taking 5 to 9 prescription medications, 65 (11.3%) receiving 10 to 14 medications, and 12 (2.0%) receiving 15 or more. Hence, the polypharmacy reported in this study is higher than in other studies. This might be due to the small study population. However, there were two studies reported that the prevalence of polypharmacy is more than 50% [35,36]. The diagnosis of central nervous system (CNS) disorders is very less in the current population as most of the elderly patients with CNS disorders are referred to specialised clinics or hospitals for further treatments; hence, the number of CNS patients in the current study is fewer.

### 4.2. Polypharmacy in Cardiovascular Diseases

Polypharmacy in this study was mostly detected with cardiovascular medications. The comorbidities identified in this study reflects the overall prevalence data collected from the Malaysian National Health and Morbidity Survey (NHMS) conducted in 2019, pointing out T2DM, hypertension, and dyslipidaemia being the major noncommunicable disease in this country [37]. As this study was carried out at primary care clinic in a rural area, complex morbidities such as dementia and osteoporosis were not detected. Patients with high morbidity burden tend to obtain specialist services at secondary or tertiary health care facilities commonly situated in the urban area. Similar to the current study findings, a number of earlier studies have reported that cardiovascular medications, particularly antihypertensives, were commonly seen in polypharmacy among the elderly population [38,39,40]. It is evident that polypharmacy has been commonly linked to cardiovascular disease. Evidence-based guidelines that recommend treatment with multiple drug classes guide polypharmacy in cardiovascular diseases. For example, in patients with very high-risk to high-risk of developing cardiovascular diseases such as stroke, statins and aspirin are routinely prescribed for primary prevention. Similar issues are also seen among elderly patients with heart failure (HF), where polypharmacy is more prevalent due to medications for both HF and non-HF comorbidities [41]. This is because clinical practice guidelines for multidrug HF regimens have unique considerations, given that they improve outcomes and symptoms of HF.

The ALLHAT trial found that amlodipine has a risk of falls among the elderly during the first year of follow-up compared to chlorthalidone (hazard ratio: 2.24; *p* = 0.03) or lisinopril (hazard ratio: 2.61; *p* = 0.04) [25]. In the United States of America, hypertension is more common in two-thirds of geriatrics (≥65 years), and the prevalence of hypertension is increased from 27.3% to 74.0% in individuals of <60 years and >80 years, respectively. The study further added that the use of blood pressure medications on a regular basis does not increase the risk of falling, whereas the risk of falls is higher during the first 24 h after initiating the antihypertensive medications [42].

### 4.3. Polypharmacy and Risk of Falls in Geriatrics

In the present study, most of the patients were on polypharmacy, and the majority were receiving fall-risk-increasing drugs (FRIDs), especially antihypertensives and antidiabetic medications. Several studies mentioned that cardiovascular and antidiabetic medications increase the risk of falls [43,44,45]. The risk of falls among the elderly is associated with various intrinsic and extrinsic factors [46]. The intrinsic factors associated with fall risk are ageing and age-related pharmacokinetic and pharmacodynamic changes that have a direct effect on one’s medications. The extrinsic factors associated with fall risks are environment, for example, how the medicines were taken at home [46,47]. Among the elderly, the prescribed medications are the potential major contributor to falls. According to WHO, adults aged 65 and above are more susceptible to multimorbidity and multiple medications and also have a higher risk of falls [48]. Multimorbidity and polypharmacy can double the risk of falls, especially the medication-related falls, when the patient receives at least one established FRID as part of the patient’s daily regimen [49].

According to the American Heart Association, the falls or risks for falling is 40% to 60% among older adults with cardiovascular disease. The risk of falls and fall-related injuries are greatly associated with their medications, the presence of heart disease, postural hypotension, and arrhythmias. The abnormalities in gait and balance, presence of physical frailty, sensory impairment, and environmental hazards also contribute to a greater number of falls and fall-related injuries [44]. The risk of falling is higher among older adults, and falls are the most common cause of injury among the elderly population. The risk of falling increases with age and the existence of both cardiovascular and non–cardiovascular disease conditions. Even though, cardiovascular medications are well tolerated initially, they can contribute to falls, and the risk of falls is higher when the elderly receive concomitant administration of medications that lower blood pressure or impair gait and balance [50].

Polypharmacy, or the use of multiple medications, is common in older patients with cardiovascular disease, who frequently have comorbidities. Depending on the clinical situation, polypharmacy may or may not be appropriate, but it is associated with an increased risk of falls. Cardiovascular medications, such as antihypertensive agents and loop diuretics, can cause falls due to hypotension and orthostatic hypotension (OH). Nonselective and mixed β-blockers are associated with an increased risk of falls when compared to selective β-blockers [51,52].

### 4.4. Diuretics in Geriatrics

In the present study, about 22.9% of the patients are on diuretics. Diuretics are often prescribed when two or more antihypertensive agents are inadequate to control high blood pressure. A study from Japan found that 16.5% of its prescriptions were prescribed with multiple diuretics [53]. Similar to this study, calcium-channel blockers and angiotensin receptor blockers were first-line agents in treating hypertension among the elderly population due to their safety profile. The number of patients diagnosed with heart failure is less in this study. Diuretics are less likely to be prescribed among the very elderly population due to electrolyte imbalance [54]. Furthermore, loop diuretics should be consumed in the morning because frequent urination at night leads to sleep disturbance, tiredness, and eventually leading to geriatric syndrome. As the risk of fall increases with age, caution need to be exercised when prescribing diuretics among the elderly population. A study conducted in the US found that the risk of falls is up to 21 days when using diuretics. Diuretics-associated volume depletion and increased excretion of calcium might increase the risk of falling and fall-related fractures among the elderly, as diuretics cause decreased bone mineral density, orthostatic hypotension, and hypocalcaemia [42].

Diuretics and SGLT2 inhibitors have the potential to cause a variety of fall-related adverse effects. Diuretics appear to have more potential fall-related adverse effects than SGLT2 inhibitors [55]. For example, prolonged use of diuretics may cause a severe electrolyte imbalance, which increases the chances of falls. Whereas the electrolyte imbalance and fall risk are lower in SGLT2 inhibitors use [56]. In general, among all the diuretics classes, loop diuretics have more fall risk [57]. The use of loop diuretics in elderly with impaired gait performance or musculoskeletal disorders doubles the risk of falls. The patient-specific characteristics and fall-related side effect profiles of drugs play a major role in the selection of the most appropriate drug regimen for elderly patients.

### 4.5. Prescribing Cascade

Prescribing cascade occurs when an additional drug is prescribed to a patient to treat an iatrogenic-induced condition caused by the initial drug [58]. The misconception is that unaware of an adverse drug reaction or side effect caused by the initial drug, it is often diagnosed as a separate medical condition. In this study, the loop diuretic, furosemide, was detected in 11 prescriptions. Out of 11 prescriptions, 8 prescriptions have amlodipine coprescribed. A meta-analysis of randomised, placebo-controlled trials reported that amlodipine causes a three-fold risk of developing peripheral oedema compared to placebo [59]. Furosemide is often prescribed to treat oedema. This is indicative of a possible prescribing cascade. Prescribing cascade is a serious problem among the elderly population because the use of multiple medications may lead to the onset of drug-related issues, which may be misdiagnosed as new geriatric syndromes such as dizziness, falls, and incontinence [60,61].

An earlier study examining the prescribing cascades among older adults with dementia revealed that prescribing cascade was commonly seen in the combination of diuretics following calcium channel blockers (CCB). There were 289 cases of a diuretic following CCB reported with prescribing cascade in the cohort of older adults [62].

A recent report on prescribing cascade in an older adult with mental health issues revealed that the older person was prescribed oral furosemide 40 mg once daily dosing for bilateral lymphoedema. Two weeks later, he was admitted to the emergency department with a recent fall and a confused mindset for the previous two weeks. Upon examination, it was noted that the patient was prescribed valproate semisodium following lithium toxicity and experiencing confusion secondary to valproic acid. It was misdiagnosed, and benzodiazepines were added to treat confusion instead of switching valproate semisodium to some other alternatives; finally, the patient ended up with a fall-related injury [63]. Hence, urgent attention is required to analyse the medications being consumed by the patients.

The current research focuses mainly on the use of medications among geriatric patients with noncommunicable diseases in a primary care setting. Comparing the current research with recent research carried out in Malaysia by Chang et al. [64], the study focused only on elderly patients with COVID-19 infections. Moreover, the previous study was conducted at tertiary care hospitals where the patients can avail advanced treatments. In contrast, the current study was conducted in a primary care setting as the primary care settings in Malaysia are almost neglected/least focused by the health authorities. Moreover, it provides only limited options for treatment, and most of the illnesses in primary settings are maintained or treated by the available medications. Hence, polypharmacy is a major concern among elderly patients who seek treatment in these primary care settings. The elderly population in this study has multimorbidity and receives multiple medications (mostly 4 or more). Polypharmacy is not only posing an economic threat to the elderly, but it also increases the chance of getting falls among elderly patients. Hence, the study suggested the health fraternity look for medication optimisation and deprescribing to reduce the future complications associated with polypharmacy.

The drugs acting on the central nervous system, cardiovascular system, endocrine and reproductive systems have a higher potential to cause impairment of gait performance, drowsiness, syncope, dizziness, electrolyte imbalance, postural hypotension, and other related complications that may end up in increasing the risk of falls and fall-related injuries. The selection of appropriate drugs for the management of clinical conditions in the elderly may reduce the risk of falls. Polypharmacy was identified as the major risk factor of falls; hence, optimising the choice of medications, appropriate dosing and dosing interval, deprescribing, regular monitoring of patient’s vital parameters, and adjusting the dose of therapy based on vital signs are paramount in the reduction in fall-related injuries among the geriatrics. Prescribers, pharmacists, patients, and allied healthcare staff can play a vital role in risk reduction strategies. The use of treatment guidelines approved by WHO, respective government bodies or the Ministry of Health, and pharmacy boards will ease the process of medicine optimisation. Enhancing awareness among the general public, elderly patients who are at risk of falls, and patient caregivers about the patient’s clinical conditions, potential and possible drug–drug or drug-food interactions, side effects, adherence to medication regimen, and follow-up visits will reduce the drug-related complications and institutionalisation.

In Malaysia, the role of clinical pharmacists in medicine optimisation has not been fully established. Most of the time, the patient and the patient’s caregivers rely on prescribers for any information related to the patient’s medical condition, medication-related information, and product counselling. Pharmacists can play a vital role in the medicine recommendation, selection of appropriate dose and frequency, and providing essential information on products, side effects, and when to seek immediate medical attention. Implementation of pharmacist-led services will reduce the risk of drug-related complications and improve patients’ adherence to their treatment regimen. A combined effort from all health care providers is essential to improve the quality of services and reduce the risk of falls and fall-related injuries among the geriatric population.

Periodical and regular awareness programmes for the elderly on the appropriate use of prescribed medications and general awareness of falls and fall risk will minimise the prevalence and incidence of drug-related harms. In addition to that, continuous professional development (CPD), continuous professional education (CPE), and periodical training programmes for prescribers and health workers will enhance the quality of service delivered to the patient’s community.

There are some limitations in the current study, as the study was conducted in a single primary care setting of the rural region of Negeri Sembilan using a convenience sampling technique; the findings of this study may not represent the whole elderly population of Malaysia. The information utilised in the study was extracted from the patient’s medical record, medication chart, and case notes, and there may be some inaccuracies in the data captured in the medical records. This may be another limitation of this current study.

## 5. Conclusions

The prevalence of polypharmacy in the current study is up to 51%. The majority of the elderly were prescribed four or more medications. Polypharmacy was commonly seen in cardiovascular drugs. Cardiovascular medications are an important part of fall-risk-increasing drugs (FRIDs), which contribute to a greater number of falls and fall-related injuries among the elderly. Hence, urgent attention is required to reduce the prevalence of polypharmacy and its related harms. Strategies such as deprescribing and dose optimisation prior addition of the drug need to be considered among the elderly population.

## Figures and Tables

**Table 1:** Demographic characteristics of the respondents (*n* = 310).

Sociodemographic Characteristics	Frequency (%) (*n* = 310)	Mean (SD)
**Sex**		
Female	169 (55)	
Male	141 (45)	
**Age (years)**		
65–69	171 (55.15)	69.72 (2.85)
70–74	104 (33.57)	
75–79	29 (9.35)	
80–84	6 (1.93)	
≥85	0	
**Ethnicity**		
Malay	308 (99)	
Chinese	0	
Indian	2 (1)	
**Education**		
No formal education	12 (4)	
Primary education	229 (74)	
Secondary Education	69 (22)	
**Marital Status**		
Married	217 (70)	
Widowed	94 (30)	
Single	0	
Divorced	0	
**Allergies**		
Yes	14 (5)	
No	296 (95)	

**Table 2:** Respondents’ comorbidity status (*n* = 310).

Diseases	Frequency	Percentage (%)
Type 2 diabetes mellitus (T2DM) + hypertension + dyslipidaemia	139	44.84
Hypertension + dyslipidaemia	87	28.06
T2DM + hypertension	15	4.84
T2DM + dyslipidaemia	11	3.55
T2DM + hypertension + dyslipidaemia + myocardial infarction (MI)	8	2.58
Dyslipidaemia	7	2.26
Chronic obstructive pulmonary disease (COPD) + hypertension + dyslipidaemia	5	1.61
Hypertension	5	1.61
T2DM + hypertension + dyslipidaemia + asthma	4	1.29
Hypertension + dyslipidaemia + MI	3	0.97
Hypertension + dyslipidaemia + gout	2	0.65
Hypertension + dyslipidaemia + asthma	2	0.65
T2DM+ dyslipidaemia + hypertension + gout	2	0.65
T2DM+ hypertension + epilepsy	2	0.65
MI + hypertension	2	0.65
T2DM + hypertension + dyslipidaemia + COPD	2	0.65
COPD	2	0.65
Chronic kidney disease (CKD) + hypertension + dyslipidaemia + gout	1	0.32
COPD + hypertension	1	0.32
T2DM + hypertension + dyslipidaemia + depression	1	0.32
Stroke + hypertension + dyslipidaemia	1	0.32
Atrial fibrillation + hypertension + dyslipidaemia	1	0.32
T2DM + hypertension + dyslipidaemia + hyperthyroidism	1	0.32
Dyslipidaemia + MI	1	0.32
T2DM + hypertension + dyslipidaemia + epilepsy	1	0.32
T2DM + hypertension + dyslipidaemia + hypothyroidism	1	0.32
Myocardial infarction	1	0.32
Hypertension + epilepsy	1	0.32
COPD + dyslipidaemia	1	0.32
Total	310	100

**Table 3:** Monotherapy and combination therapy prescribed to the respondents (*n* = 310).

Drug Therapy	Frequency	Percentage (%)
**Monotherapy**	**8**	**2.58**
Simvastatin	4	50.00
Amlodipine	3	37.50
Atorvastatin	1	12.50
**Combination therapy**	**302**	**97.42**
List of top 10 combination therapy		
Amlodipine + simvastatin	17	5.63
Simvastatin + perindopril + amlodipine + metformin	10	3.31
Perindopril + amlodipine + simvastatin	8	2.65
Amlodipine + simvastatin + metformin	8	2.65
Metformin + gliclazide + simvastatin + amlodipine + perindopril	6	1.99
Perindopril + amlodipine + simvastatin + mecobalamin	4	1.32
Metformin + simvastatin + amlodipine + gliclazide	4	1.32
Amlodipine + perindopril + simvastatin + aspirin	4	1.32
Perindopril + simvastatin	3	0.99
Simvastatin + amlodipine, metformin + gliclazide + perindopril + hydrochlorothiazide	3	0.99
Other combinations	235	77.81

**Table 4:** The number of prescribed medications among the respondents (*n* = 310).

Number of Drugs	Frequency	Percentage (%)	Mean ± SD; *p*-Value
10	1	0.33	5.18 ± 0.64; 0.007 *
9	6	1.99	
8	13	4.30	
7	23	7.62	
6	53	17.55	
5	65	21.52	
4	69	22.85	
3	40	13.25	
2	32	10.60	
1	8	2.58	
Total	310	100	

* *p* < 0.05 is considered statistically significant.

**Table 5:** Frequency of medications prescribed (*n* = 310).

Drug Classes	Drugs	Frequency (%)
Cardiovascular	Simvastatin	262 (84.51)
	Amlodipine	235 (75.80)
	Perindopril	166 (53.54)
	Aspirin	70 (22.58)
	Hydrochlorothiazide	58 (18.71)
	Losartan	31 (10.00)
	Atenolol	28 (9.03)
	Prazosin	28 (9.03)
	Bisoprolol	22 (7.09)
	Atorvastatin	15 (4.84)
	Frusemide	11 (3.55)
	Metoprolol	7 (2.26)
	Telmisartan	6 (1.94)
Endocrine	Metformin	169 (54.52)
	Gliclazide	65 (20.97)
	Insulin	57 (18.38)
Antipsychotics	Amitriptyline	1 (0.32)
Supplements	Mecobalamin	87 (28.06)
	Calcium	23 (7.42)

**Table 6:** Drug-related problems in the prescriptions.

Issues	Explanations
Prescribing Cascade	Eight out of eleven prescriptions with frusemide were prescribed with amlodipine.
Lack of treatment optimisation	A patient was prescribed a stand-alone β-agonist inhaler for asthma when a corticosteroid inhaler needs to be prescribed concomitantly.
Inappropriate prescription	Amitriptyline was prescribed for depression. Increased risk of falls and anticholinergic side effects.

## Data Availability

The data presented in this study are available on request from the corresponding author. The data are not publicly available due to ethical restrictions.
